# Diastolic dysfunction in pulmonary artery hypertension: Creatine kinase and the potential therapeutic benefit of beta‐blockers

**DOI:** 10.1111/1440-1681.12898

**Published:** 2018-01-10

**Authors:** Ewan D Fowler, Mark J Drinkhill, Rachel Stones, Ed White

**Affiliations:** ^1^ Multidisciplinary Cardiovascular Research Centre University of Leeds Leeds UK; ^2^ School of Physiology, Pharmacology & Neuroscience University of Bristol Bristol UK

**Keywords:** beta‐blockers, creatine kinase, diastolic dysfunction, heart failure, pulmonary artery hypertension

## Abstract

Passive properties of the myocardium influence diastolic filling and cardiac output. In heart failure, changes in contributors to the passive properties of the ventricle, such as titin and collagen, and loss of the metabolic enzyme creatine kinase, increase resistance to filling resulting in diastolic dysfunction. Pulmonary artery hypertension (PAH) arises from interactions between the pulmonary vasculature and the right ventricle (RV) which ultimately leads to RV failure. Beta1‐adrenergic receptor blockers (BB) act on the myocardium and are beneficial in left heart failure but are not used in PAH. We investigated whether BB improved survival and RV function in a rat model of PAH. Rats were injected with monocrotaline (60 mg/kg) to induce PAH and RV failure, or saline as controls (CON). When PAH was established, rats were treated with metoprolol (10 mg/kg per day) (MCT+BB) or vehicle (sucrose) (MCT); CON were treated with vehicle. In vivo measurement of RV compliance using pressure–volume catheter, indicated diastolic dysfunction in the RV of MCT rats was improved with BB treatment. Expression of creatine kinase protein and mRNA was lower in MCT rats compared to CON, with a trend for reversion by BB treatment. Isolated CON RV myocytes had a positive contraction response to faster pacing, whereas it was negative in MCT. MCT+BB cells had an intermediate response, indicating improved ability to respond to increased demand. BB improved diastolic function, partially restored metabolic enzymes and augmented contractility in PAH. These data support the hypothesis that BB may be beneficial in PAH by supporting RV function.

## 1 DIASTOLIC DYSFUNCTION AND HEART FAILURE

Ventricular stroke volume is influenced by end‐diastolic volume through the Frank‐Starling mechanism. This dictates that as the ventricle fills, sarcomeres are stretched along the ascending limb of the length‐tension relationship, resulting in greater myofilament force generation and ejection of blood. In pathological conditions, such as heart failure, increased resistance to ventricular filling may reduce end‐diastolic volumes and thereby cause a decrease in stroke volume and cardiac output. This limits the ability of the heart to respond to an increase in demand, which is a definition of heart failure. Myocardial passive properties are implicated in diastolic dysfunction. During normal filling, a major contribution to passive tension is thought to come from titin, an elastic protein that spans half of the sarcomere from Z‐disk to M‐line. Changes in the properties of titin in disease are known to include a shift in isoform distribution from the more compliant N2BA isoform to the shorter and stiffer N2B isoform, oxidation of cysteine residues, and phosphorylation status.^1^ The extracellular matrix also contributes to passive tension in volume‐overload situations. Proliferation of collagen fibres (fibrosis) and greater cross‐linking between fibres can increase myocardial stiffness^2^ and mechanisms involving the myofilaments and cytoskeleton may also be relevant.

In addition, metabolic state also influences cardiac passive properties. Creatine kinase (CK) catalyses the reversible transfer of inorganic phosphate from creatine phosphate to ADP, generating ATP.

$${\text{MgADP}}^{-}+{\text{PCr}}^{2-}+H^{+}↔{\text{MgATP}}^{2-}+\text{Cr}$$

In cardiac muscle there are four isoforms: CK‐m (muscle) that accounts for about 70% of total CK, of which 10%‐30% is bound to the myofilament protein myomesin; CK‐mt (mitochondrial) 20%‐30% total; CK‐b (brain) and CK‐mb (muscle/brain) are both found in low abundance but have been reported to increase in disease. CK is located in areas with high ATP turnover such as mitochondria, myofilaments, sarcoplasmic reticulum and sarcolemma.^3^ This functional coupling with ATPases helps maintain a high local ATP:ADP essential for, e.g. cross‐bridge cycling and Ca^2+^ uptake.^4^, ^5^ Indeed, a consequence of ADP accumulation is an increase in passive tension in the heart due to rigor‐like cross‐bridge formation.^6^, ^7^ CK expression decreases in left heart failure,^8^ which may have multiple repercussions in the cardiac myocyte.

## 2 PULMONARY ARTERY HYPERTENSION AND DIASTOLIC DYSFUNCTION

Pulmonary artery hypertension (PAH) is a complex disease with interactions between the pulmonary vasculature (PV) and the right ventricle (RV). It is caused by increased constriction of the PV which increases vascular resistance and hence RV afterload. This eventually leads to RV failure, which is the most common cause of death in PAH.

In the study of Rain et al^9^ human PAH patients were found to have diastolic dysfunction. There was increased stiffness of myocardium, increased collagen content and reduced phosphorylation of titin with no shift in titin isoforms. RV failure has also been studied in animal models of PAH.^10^ In the monocrotaline rat model of PAH, RV heart failure occurs 3‐4 weeks after a single injection of monocrotaline. The onset of heart failure in this model was associated with diastolic dysfunction.^11^ When a reduced dose of monocrotaline was used to induce stable hypertrophy, diastolic function was preserved.^12^

We have used the monocrotaline rat model to study RV failure in PAH. The end diastolic pressure–volume relationship (EDPVR) was measured using a Millar catheter inserted into the RV of anesthetized animals. The EDPVR was significantly steeper in monocrotaline‐injected rats (MCT; [FAIL in Fowler *et al*.^13^]) compared to saline‐injected controls (CON), indicating reduced compliance and greater resistance to ventricular filling and confirming the presence of diastolic dysfunction. This was not linked to increased fibrosis, therefore changes in the properties of single ventricular myocytes were investigated.

Intact single myocytes were attached to glass fibres and electrically stimulated to contract while being stretched. The end ‐diastolic force‐length relationship (EDFLR) was recorded and the slope was found to be significantly steeper in RV myocytes from MCT rats, in accord with the steeper EDPVR in vivo. Furthermore, it was observed that RV myocytes (but not left ventricular myocytes) from MCT rats had significantly shorter diastolic sarcomere lengths compared to CON animals, in the absence of changes in either diastolic intracellular Ca^2+^ (measured with Fura‐2) or myofilament Ca^2+^ sensitivity. Application of an intracellular Ca^2+^‐buffer (BAPTA‐AM) increased sarcomere length; subsequent addition of a myofilament cross‐bridge inhibitor (40 mmol L^−1^ BDM) caused further lengthening. The sarcomere lengthening effect of BDM was significantly greater in MCT than CON, suggesting a cross‐bridge based, Ca^2+^‐independent mechanism.

Western blot analysis showed a significant reduction in protein levels of CK in the RV of MCT rats. Furthermore, when the sarcolemma was permeabilised with saponin and cells incubated with exogenous CK and creatine phosphate, the sarcomere length of RV myocytes from MCT rats significantly increased. Conversely in CON myocytes, inhibition of CK shortened sarcomere length. This indicated that loss of CK in MCT myocytes impaired RV diastolic function. In intact CON RV myocytes, the relationship between stimulation frequency and myocyte shortening was relatively flat over the range 1‐7 Hz, in contrast the relationship in MCT myocytes was steeply negative. A similar steep negative relationship occurred in CON myocytes following CK inhibition. We interpreted this as a decreased ability of MCT myocytes to respond to an increase in demand, which is a definition of heart failure. We concluded that decreased CK expression leads to diastolic dysfunction, via local reduction in ATP:ADP ratio and thus to Ca^2+^‐independent force production and diastolic sarcomere shortening.^13^

## 3 BETA‐BLOCKER TREATMENT OF PAH

There is currently no cure for PAH and novel treatments are needed to resolve this. The importance of RV function in PAH patient survival indicates the heart is a potential therapeutic target. However, available therapies primarily cause vasodilation of the PV and current guidelines do not include treatments specifically for the RV.^14^ It was proposed that established treatments for left ventricular failure may be beneficial to the failing RV in PAH.^15^, ^16^ Beta‐adrenoceptor blockers (beta‐blockers) are a primary treatment for left ventricular failure which slow the cardiac remodelling caused by chronic sympathetic activation. Small clinical trials have tested their use in PAH without finding adverse effects.^17^

Pre‐clinical studies have shown beta‐blockers improved animal survival, vascular remodelling and cardiac function in PAH. However, the mechanisms contributing to improved cardiac function have not been investigated. We therefore proposed the first study to investigate the mechanisms by which beta‐blockade may benefit RV myocytes in PAH. From our previous findings, discussed in the preceding section, we hypothesized that beta‐blockade would improve diastolic dysfunction and the response to increased demand in monocrotaline‐treated rats, and that these changes would be associated with increased levels of CK.

Experiments were conducted with local ethical approval and in accordance with UK Home Office and European Parliament Directive 2010/63/EU guidelines on the use of animals in research. Methodological details can be found in Fowler et al^13^ PAH and RV failure were induced in rats by a single IP injection (60 mg/kg) of monocrotaline. When PAH was established, metoprolol in sucrose solution (10 mg/kg per day, MCT+BB) or sucrose solution (MCT) was administered by voluntary syringe feeding. Control animals (CON) were injected with saline and given sucrose.

As we were specifically interested in the cardiac effects of beta‐blocker treatment we chose to use metoprolol: it is beta‐1 specific (the predominant beta‐adrenoceptor in heart), in common clinical use, and lacks the vasodilatory effects of carvedilol^18^ and nebivolol.^19^ The human equivalent dose of 10 mg/kg per day was 1.62 mg/kg per day, within the typical clinical dose range for a 75 kg patient (0.17‐2.67 mg/kg per day). Animals were killed when showing external signs of heart failure (weight loss, lethargy, laboured breathing, piloerection) (see Fowler et al^13^). Data from the three experimental groups were compared by one‐way ANOVA. Holm‐Sidak (n numbers equal) or Tukey (n numbers uneven) *post hoc* tests were used following parametric ANOVA, or Dunn *post hoc* tests following non‐parametric ANOVA, as recommended by the statistical package (GraphPad Prism 7). Statistical differences are reported as *P *<* *.05.

**Figure 1:**
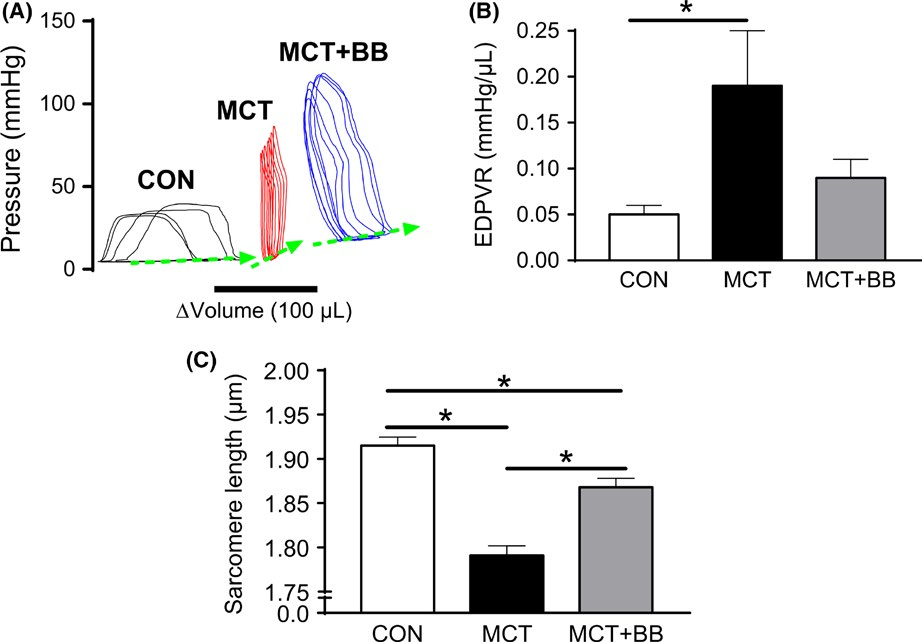
Beta‐Blocker treatment improves EDPVR and increases resting sarcomere length. (A), PV relationships from a CON (black), MCT+BB (blue) and MCT (red) animal. The EDPVR was recorded during progressive vena cava occlusion. The loops from the three groups are displaced along the *x*‐axis and averaged for clarity. (B), There was a significantly steeper EDPVR in MCT (N = 7 animals) than CON (N = 10 animals) (*P* < .05) indicating diastolic dysfunction in MCT. The relationship for MCT+BB (N = 5 animals) was intermediate indicating an improvement in diastolic function. One‐way ANOVA with Tukey *post hoc* test. (C), In isolated RV myocytes resting sarcomere length progressively shortened CON > MCT+BB > MCT (n = 30 cells from N = 6 rats per group). * *P* < .05, one‐way ANOVA with Holm‐Sidak *post hoc* test. CON, control; MCT, monocrotaline treated; MCT+BB, monocrotaline + beta‐blocker treated; EDPVR, end‐diastolic pressure‐volume relationship

Treatment with metoprolol delayed the median time to the onset of heart failure signs from 23 days post MCT (n = 12) to 31 days (n = 15) (*P* < .01 Mantel‐Cox test). To assess the mechanisms of BB action, MCT animals were compared to CON and MCT+BB animals at 23 ± 1 days after a single injection of saline or MCT. Ventricular weights are given in Table 1 and show RV hypertrophy in both PAH groups. EDPVRs were measured in anaethetised rats by Millar catheter in the RV during vena cava occlusion (Figure 1A, see Fowler *et al*.^13^ for details of methodology). The EDPVR from MCT animals was significantly steeper than CON, indicating diastolic dysfunction. The mean value for MCT+BB was intermediate (Figure 1B) representing an improvement in diastolic function. Consistent with observations in whole animals, the resting sarcomere length of RV myocytes showed a progressive increase CON > MCT+BB > MCT (Figure 1C). BB treatment did not affect diastolic or systolic pulmonary pressure, consistent with previous reports using other BB^18^, ^19^, but did improve the ratio of end systolic pressure–volume relationship (ESPVR) to arterial elastance (Ea), an indicator of in vivo contractility/afterload (see Table 1).

**Table 1:** Animal and organ characteristics of rats on the median day of heart failure signs (CON and MCT+BB) or day of heart failure signs (MCT)

	CON	MCT	MCT±BB
Rats/group	15	13	14
Organ weights			
Body weight (g)	301 ± 6	257 ± 4^a^	274 ± 8^a^
Heart/body weight (mg/g)	3.65 ± 0.16	4.82 ± 0.23^a^	5.18 ± 0.30^a^
Lung/body weight (mg/g)	4.76 ± 0.39	8.74 ± 0.46^a^	9.72 ± 0.37^a^
RV/body weight (mg/g)	0.64 ± 0.06	1.20 ± 0.06^a^	1.14 ± 0.06^a^
LV+S/body weight (mg/g)	2.20 ± 0.10	2.55 ± 0.13	2.58 ± 0.09^a^
RV:LV+S ratio	0.30 ± 0.04	0.48 ± 0.03^a^	0.45 ± 0.02^a^
Rats/group	9	7	5
In vivo			
End systolic pressure (mm Hg)	39.5 ± 2.7	83.2 ± 5.2^a^	101.8 ± 9.9^a^
End diastolic pressure (mm Hg)	4.6 ± 0.7	9.6 ± 0.7^a^	9.9 ± 2.3^a^
Stroke volume (μL)	110 ± 10	48 ± 10^a^	65 ± 5^a^
ESPVR/Ea	0.93 ± 0.27	0.27 ± 0.06^a^	0.57 ± 0.20
EDPVR (mm Hg/μL)	0.05 ± 0.01	0.19 ± 0.06^a^	0.09 ± 0.02

On the final experimental day there was RV hypertrophy in MCT and MCT+BB animals compared with CON. RV systolic and diastolic pressure were increased in MCT and MCT+BB animals. Ventriculo‐arterial coupling was impaired in MCT (reduced end‐systolic pressure–volume relationship (ESPVR) to arterial elastance (Ea) ratio) but not different in MCT+BB compared to CON.

a *P* < .05, one‐way ANOVA. Intergroup differences between variables were identified using Dunn's (RV:LV+S and End systolic pressure) or Tukey's (all others) *post hoc* test.

CON, control; MCT, monocrotaline treated; MCT+BB, monocrotaline + beta‐blocker treated; LV, left ventricle; RV, right ventricle; S, septum

**Figure 2:**
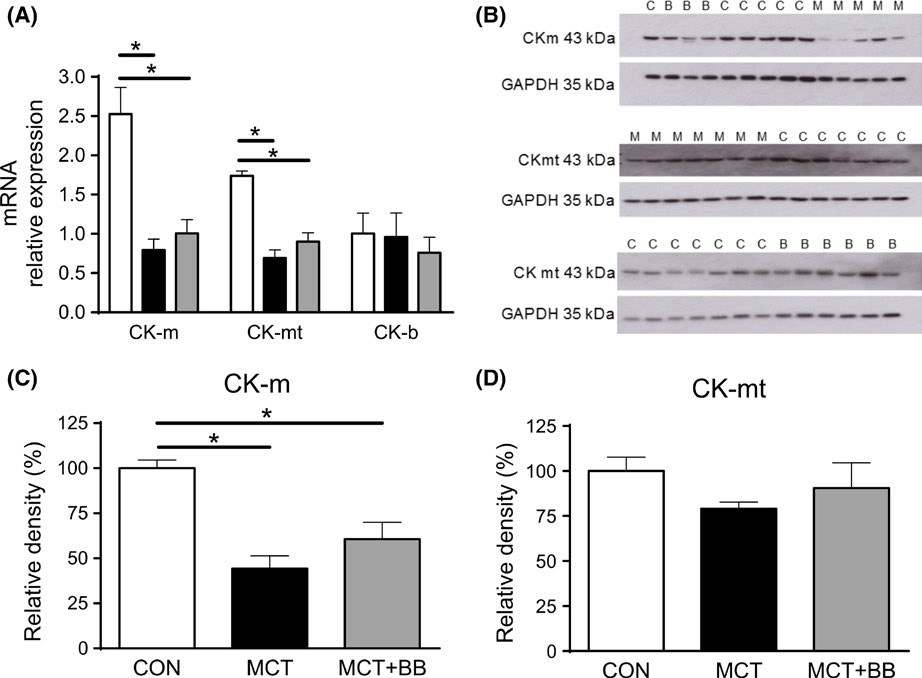
Loss of creatine kinase was attenuated by BB treatment. (A), Creatine kinase (CK) isoenzyme distribution in cardiac muscle consists of 70% muscle (CK‐m), 20% mitochondrial (CK‐mt) and the remainder brain (CK‐b) isoforms. Expression of mRNA for the predominant CK isoforms were reduced in MCT+BB and MCT rats compared to CON. Mean values for MCT+BB were greater than MCT but not statistically so. N = 10 rats per group. One‐way ANOVA with Holm‐Sidak *post hoc* test. (B), Western blots for CK‐m and CK‐mt. C, CON; B, MCT+BB; M, MCT. Common CON samples were run on each gel as calibrator samples where N precluded use of a single gel. Blots were stripped and re‐probed for GAPDH hence changes in background density. Protein levels for (C), CK‐m (N = 6‐10 rats per group) were decreased in MCT compared to CON, levels for MCT+BB were intermediate. One‐way ANOVA with Dunn's *post hoc* test. (D) A similar trend was observed in CK‐mt expression, although differences were not statistically significant (N = 6‐7 rats per group). Protein levels were normalised to GAPDH and expressed relative to the mean value in CON. *P < 0.05 CON, control; MCT, monocrotaline treated; MCT+BB, monocrotaline + beta‐blocker treated

Our previous observations had linked CK to these changes, Figure 2 shows the levels of mRNA for CK‐m and CK‐mt (Figure 2A) were significantly reduced in MCT vs CON. Protein levels for CK‐m (Figure 2B, C) were also significantly lower in MCT. Consistent with our previous report, CK‐mt tended to be reduced in MCT by around 20% (Figure 2D). There were no significant differences in the levels of CK‐m and CK‐mito, mRNA or protein levels between MCT+BB and MCT. However, in each of the four comparisons, mean values for MCT+BB were intermediate between CON and MCT indicating a trend for improvement. Furthermore, we have presented data for CK mRNA and protein expression, but this is not always synonymous with enzyme activity, particularly if the *in situ* environment in failing hearts has increased oxidative stress^20^ or reduced substrate availability, both of which are thought to decrease CK activity. It is interesting to note that the use of beta‐blockers to improve CK function is consistent with previous findings in a model of myocardial infarction.^21^

**Figure 3:**
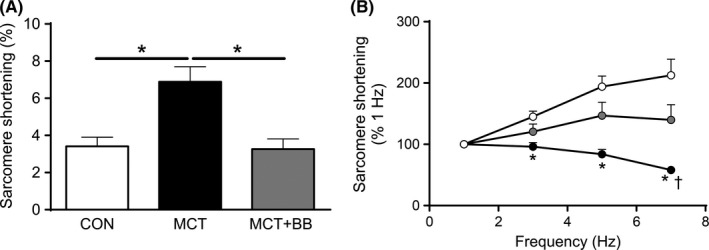
Beta‐blocker treatment partially restored the profile of isolated RV myocyte contraction–frequency relationships. (A), Unloaded sarcomere fractional shortening was recorded in isolated cells electrically paced at 1 Hz. Shortening was greater in MCT than CON and MCT+BB (*P < .05, one‐way ANOVA with Tukey's *post hoc* test). (B), Shortening normalised to values at 1 Hz. When stimulation frequency was increased to 7 Hz CON myocytes showed a positive response whereas the response in MCT was negative. The response of MCT+BB was intermediate demonstrating an increased ability to maintain contraction amplitude in response to the increase in demand (n = 22‐30 myocytes from N = 6 rats per group). Only cells which contracted regularly at 7 Hz were included for analysis. *P < .05 *vs* CON, ^†^ *P* < .05 *vs* MCT+BB. Within‐group response to frequency was assessed using one‐way repeated measures ANOVA. Between‐group differences at each frequency were identified using one‐way ANOVA with Dunn's *post hoc* test while accounting for multiple comparisons using the Bonferroni method CON, control; MCT, monocrotaline treated; MCT+BB, monocrotaline + beta‐blocker treated

At low stimulation frequency (1 Hz) MCT myocytes had greater sarcomere shortening (Figure 3A) than CON and MCT+BB, which we believe is related to the longer action potential duration in MCT myocytes promoting greater loading of Ca^2+^ stores.^22^ However, when demand was increased by increasing stimulation frequency, CON myocytes showed a positive or flat contraction–frequency relationship compared with a negative relationship in MCT. Myocytes from beta‐blocker treated rats had an intermediate relationship, such that fractional shortening was not different to CON at any frequency and was significantly greater than MCT at 7 Hz.

## 4 CONCLUSIONS

Data for CON and MCT groups confirm the findings from our previous study.^13^ In addition, we show that parameters modulated by PAH consistently show means intermediate to CON and MCT following beta‐blocker therapy. This suggests beta‐blocker treatment may contribute to delaying the onset of heart failure by reducing maladaptive cellular remodelling within the RV. That these effects on the myocardium were achieved in the absence of improvement in lung weights or pulmonary artery pressure suggest they are due to direct effects on the myocardium. This interpretation would support the use of beta‐blockers as an adjunct to, rather than a replacement for, conventional treatments for PAH. Despite increasing pre‐clinical evidence supporting a beneficial effect and revealing their mode of action, the use of beta‐blockers to treat PAH is at present controversial,^17^ and further data from clinical trials are required.

## ACKNOWLEDGEMENTS

This work was supported by the British Heart Foundation and a University of Leeds Ph.D. studentship to Ewan D. Fowler.
